# Construction of a COI DNA barcode reference library for non-volant terrestrial mammals of Taiwan, with notes on the effects of nuclear mitochondrial pseudogenes (numts) on molecular identification

**DOI:** 10.3897/zookeys.1293.190114

**Published:** 2026-09-24

**Authors:** Yen-Jean Chen, Yi-Lun Chiang, Meng-Jou Chi, Shih-Wei Chang, Nian-Hong Jang-Liaw

**Affiliations:** 1 National Museum of Natural Science, Taichung, Taiwan National Museum of Natural Science Taichung Taiwan; 2 Wildone Wildlife Conservation Association, Taitung, Taiwan Wildone Wildlife Conservation Association Taitung Taiwan; 3 Taiwan Biodiversity Research Institute, Ministry of Agriculture, Nantou, Taiwan Taiwan Biodiversity Research Institute, Ministry of Agriculture Nantou Taiwan; 4 Taipei Zoo, Taipei, Taiwan Taipei Zoo Taipei Taiwan

**Keywords:** DNA barcoding, cytochrome c oxidase subunit I, Barcode Index Number, conservation forensics

## Abstract

DNA barcoding using mitochondrial COI is widely applied for animal species identification, but its accuracy in mammals can be affected by uneven sampling, sequence artefacts, and nuclear mitochondrial pseudogenes (numts). In this study, we established a voucher-linked COI DNA barcode reference library for non-volant terrestrial mammals of Taiwan and evaluated its identification performance and potential limitations using the BOLD workbench, Barcode Index Number (BIN) assignments, barcode gap analyses, and comparisons with public sequence data. A total of 157 newly generated COI sequences were obtained, representing seven orders, 16 families, 37 genera and 48 species. Among the 48 sampled taxa, 15 (31.25%) had no COI barcode record available in BOLD before this study; therefore, the deposition of the present library increasing BOLD coverage for the sampled taxa from 68.8% to 100%. In the original BOLD project dataset, 145 of the 157 sequences were assigned to 49 BINs. To evaluate whether the observed barcode patterns were robust for broader sampling, 75 public Taiwanese mammalian COI sequences were incorporated into an expanded Taiwan dataset of 232 sequences. Barcode-gap and Neighbor-Joining analyses showed that 45 of the 48 taxa retained positive barcode gaps after public sequences were added, supporting the utility of COI barcodes for identifying most non-volant terrestrial mammals in Taiwan. Three taxa, *Mogera insularis*, *M. kanoana*, and *Petaurista grandis*, showed negative barcode gaps and/or split clustering patterns and were therefore highlighted for further investigation. In addition, all three newly generated *Rattus tanezumi* sequences were flagged as putative numts based on anomalous placement and full-length sequence inspection. Our results further emphasize the importance of integrating specimen traceability, functional sequence checks, comparisons with public data, distance-based analyses, and cautious interpretation of BIN results in mammalian DNA barcoding research.

## Introduction

The mitochondrial cytochrome c oxidase subunit I (COI) gene has been standardized and adopted as a universal animal DNA barcode (Hebert et al. 2004), greatly improving the speed and accuracy of species identification. During the past two decades, DNA barcoding has become an important tool in mammalian taxonomy and conservation research, and a complete, carefully curated DNA barcode library is critical for modern conservation biology (Ratnasingham and Hebert 2007), especially in Asia, where species diversity and the proportion of cryptic species are high. Mitochondrial COI sequences have been effectively used for species identification and analyses of genetic divergence in Asian mammals, including bats in Malaysia and India, ungulates from the Indian subcontinent, and coastal cetaceans (Francis et al. 2010; Wilson et al. 2014; Kusuminda et al. 2022). Samples were obtained from field collections, museum specimens, and public databases [e.g., the Barcode of Life Data System (BOLD) and GenBank]. Most studies employ PCR amplification and Sanger sequencing and use neighbor-joining trees or Bayesian approaches for genetic distance and clustering analyses (Kumar et al. 2017; Bhaskar et al. 2020). These studies indicate that COI barcodes can discriminate most mammal species and have revealed multiple potential cryptic species, particularly in bats and cervids, suggesting that mammalian diversity in Asia remains underestimated (Francis et al. 2010; Kusuminda et al. 2022). Barcode data have also helped clarify genetic differentiation among ungulate populations in India and Southeast Asia, providing evidence for defining conservation units (Bhaskar et al. 2020). Several studies have further demonstrated the utility of DNA barcoding in wildlife trade monitoring and identification of threatened species, contributing to the implementation of CITES and forensic identification (Chang et al. 2014; Wilson et al. 2016; Kumar et al. 2017; Kundu et al. 2019). These advances improve the efficiency of Asian mammal taxonomy and monitoring and promote the establishment of regional barcode databases, laying the foundation for subsequent ecological and evolutionary research. Increasing concerns about food safety and the international smuggling of wildlife products also underscore the practical value of molecular identification in wildlife forensics, enabling enforcement personnel to rapidly and accurately identify protected or CITES-listed species from highly processed or fragmented samples (e.g., meat, fur, or ingredients of traditional medicines; Chang et al. 2014, 2018; Mwale et al. 2017; Wang et al. 2024). In addition, these resources play a foundational role in validating and interpreting animal ecological studies, such as non-invasive molecular scatology approaches that analyze predator feces to reconstruct food webs (Deagle et al. 2009; Jang-Liaw 2021).

To improve the comparability and standardization of COI barcode data, BOLD further developed the Barcode Index Number (BIN) system, which automatically clusters sequences into operational taxonomic units (OTUs) using distance-based algorithms to support preliminary assessments of species boundaries (Ratnasingham and Hebert 2013). Nevertheless, the effectiveness of DNA barcoding is not without limitations across all taxa, and its accuracy is influenced by sample quality, taxonomic frameworks, sampling density, and molecular evolutionary characteristics (Krishna Krishnamurthy and Francis 2012; Collins and Cruickshank 2013; Ratnasingham and Hebert 2013). In mammals, COI barcoding generally shows strong species identification performance; however, the presence of nuclear mitochondrial pseudogenes (numts) can lead to sequence misinterpretation, misplaced relationships, and potentially affect the inference of species boundaries (Song et al. 2008). Pseudogene-related problems have also been reported in DNA barcoding studies from Taiwan; for example, Chen et al. (2024) documented COI-like pseudogene sequences in a Taiwanese Carnidae dataset and highlighted potential challenges in barcode amplification and interpretation. Mammalian examples of numt-related problems have been reported or explicitly addressed in rodent and carnivoran datasets, including COI-based barcoding of the rodent genus *Oligoryzomys* and mitochondrial genome reconstruction in *Panthera* (da Cruz et al. 2019; de Flamingh et al. 2023). However, taxon-specific information on COInumts remains limited for most Taiwanese non-volant terrestrial mammals examined in this study. Therefore, suspicious sequences in a regional barcode library should be evaluated using multiple lines of evidence, including translation-based screening, tree placement, distance analyses, barcode-gap comparisons, and cautious interpretation of BIN assignments. Numts are non-functional copies that result from the transfer of mitochondrial DNA to the nuclear genome and often contain frameshifts or stop codons, yet they may still be co-amplified during PCR (Bensasson et al. 2001).

Taiwan is an island situated off the southeastern coast of Eurasia, spanning approximately 21°54' to 25°18'N latitude. The island’s striking topographic heterogeneity creates dramatic climatic zonation, ranging from tropical lowlands to alpine peaks, resulting in an exceptionally high level of biodiversity, making it a globally recognized hotspot (Ye et al. 2023; Shao et al. 2025). The terrestrial mammal fauna of Taiwan includes charismatic large mammals, such as the Formosan black bear *Ursus thibetanus formosanus*; ungulates, such as sambar deer; diverse small carnivores, such as the leopard cat *Prionailurus bengalensis*, crab-eating mongoose, and masked palm civet, as well as numerous endemic bats, shrews, and rodents (Qi 2008; Huang et al. 2020; Cheng et al. 2024). The cryptic nature and morphological plasticity of certain taxa (e.g., *Niviventer* and *Apodemus* species) frequently complicate traditional taxonomic and ecological investigations, highlighting the need for a precise molecular identification system (Ge et al. 2018; Liu et al. 2022; Pan et al. 2026).

The collections of the National Museum of Natural Science (**NMNS**) constitute Taiwan’s largest natural history repository and provide data with historical and geographical dimensions beyond a single time point. These holdings include more than one million specimens of plants, insects, amphibians, reptiles, and mammals, most of which are accompanied by precise collection dates and locality information, forming an indispensable foundation for biodiversity research in the country. NMNS collections are particularly critical for mammalogy because they preserve voucher specimens essential for clarifying Taiwan’s natural history (Kawada et al. 2007; Lin et al. 2010; Chen et al. 2017, 2021; Oshida et al. 2025; Tsutaya et al. 2025). The Cryobank also stores a large number of animal tissues and DNA samples, providing valuable materials for molecular genetic studies and opportunities to investigate the species identification, evolutionary history, and ecology of Taiwanese wildlife (Watanabe et al. 2007; Jang-Liaw et al. 2008, 2025; Jang-Liaw and Lee 2009; Jang-Liaw and Chou 2011). In short, NMNS collections are not only materials for scientific research but also time capsules documenting ecological change and safeguarding Taiwan’s natural heritage. The Taipei Zoo has long served as a government-designated wildlife rescue center in northern Taiwan. Tissues (muscle, liver, or blood) from captive or rescued animals are collected after pathological examinations of dead or diseased individuals and preserved in liquid nitrogen at the Taipei Zoo Cryobank (**TZC**). Furthermore, collaborations with NGOs, such as the WildOne Taiwan Wild Animal Conservation Association, in wildlife rescue missions have expanded the sources of samples in the TZC. These samples were ideal for this study (Jang-Liaw et al. 2025).

Although Taiwan has launched the Taiwan Barcode of Life initiative (TaiBOL; Chang et al. 2017; Shao et al. 2025), a comprehensive and publicly available genetic reference for terrestrial mammals on the island remains a priority. This study focused on non-volant terrestrial mammals of Taiwan and primarily used vouchered mammal collections from the NMNS, supplemented with muscle tissue samples archived in the TZC from rescued animals. We established a voucher-linked DNA barcode reference library for terrestrial mammals in Taiwan in BOLD, providing a foundational COI dataset to fill this critical gap while evaluating identification performance and exploring the potential effects of numts on molecular identification and diversity estimation in Taiwan. To address the limitation of uneven and often low sample sizes per species, we incorporated public Taiwanese mammalian COI sequences available from existing sequence databases into an expanded dataset and used them to reassess barcode gaps, clustering patterns, and suspicious lineages. Bats (Chiroptera) were excluded from this study because Taiwan harbors a large number of bat species (up to 38 species, including offshore islands such as Penghu, Kinmen, and Matsu; Huang et al. 2020), and currently, the number of species with available tissue samples suitable for DNA research is insufficient.

## Materials and methods

### Sample collection

**Figure 1.**
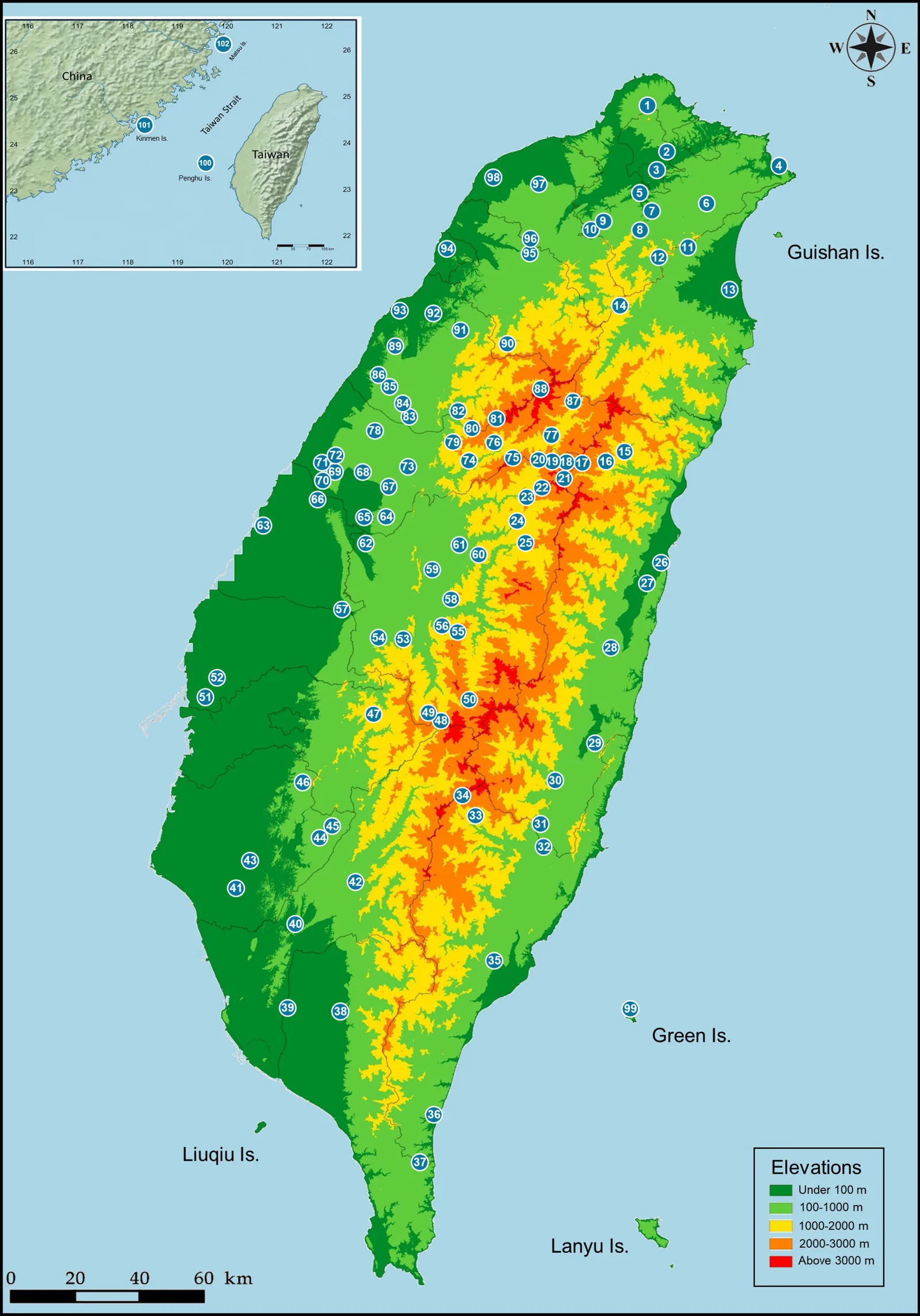
Geographic distribution of sampling localities represented by the 157 newly generated COI barcode sequences of non-volant terrestrial mammals of Taiwan. Blue circles indicate locality records compiled from specimen metadata, and numbers correspond to locality codes listed in Table 1 and Suppl. material 1. Elevation is shown in five classes: less than 100 m, 100–1000 m, 1000–2000 m, 2000–3000 m, and above 3000 m. The inset map shows the relative positions of Taiwan and offshore island localities, including Penghu, Kinmen, and Matsu.

A total of 157 tissue samples were used in this study, representing 48 confirmed species of non-volant terrestrial mammals from seven orders, 16 families, and 37 genera (Table 1). The clouded leopard *Neofelis nebulosa brachyura* (Swinhoe, 1863) has been confirmed to be extinct in Taiwan (Chiang et al. 2015) and was not included. The Eurasian otter is aquatic, and its last record on the main island of Taiwan dates back to approximately 1974 (Jang-Liaw et al. 2023). The population on the main island is likely extirpated, and the samples used in this study were from wild individuals on Kinmen Island, adjacent to Fujian Province in China. Among the tissue samples, 129 were obtained from vouchered specimens deposited in the NMNS, ensuring taxonomic accuracy, and the remaining 28 samples were from genetic tissue samples of rescued animals archived in the TZC, increasing the number of replicates in this study. The taxonomic status of each specimen was confirmed by expert curation, mainly adhering to the latest nomenclature of Taiwanese mammalian fauna (Cheng et al. 2024). Almost all samples have physical vouchers and are linked to catalogue numbers, collection localities, and identification information (Suppl. material 1). Sampling localities were compiled from specimen metadata and mapped to provide an overview of the geographic coverage of the newly generated barcode library (Fig. 1).

**Table 1.** Species composition and sample sizes of non-volant terrestrial mammals newly sequenced and analyzed in this study. The dataset comprises 157 newly generated COI sequences representing 48 recognized species from seven orders, 16 families, and 37 genera. Species numbers correspond to those used in Figs 2, 3, 4 and Suppl. materials 3–5. Sampling locality codes correspond to the geographic records shown in Fig. 1 and the detailed specimen metadata provided in Suppl. material 1. Successful COI primer sets are listed for each species. Public GenBank sequences incorporated into the expanded Taiwan dataset are listed separately in Suppl. material 2.

**Species No**.	**Order**	**Family**	**Species**	**Nominal names**	**Corresponding scientific names on BOLD**	Chinese names	**n**	**Primers**	**Institution Storing**	**Location in Fig. 1**	**Note**
1	Primates	Cercopithecidae	*Macaca cyclopis*	Swinhoe, 1863		臺灣獼猴	4	FishF1/R1	TPZ, NMNS	15, 16, 60 (2)	Species endemic to Taiwan
2	Eulipotyphla	Soricidae	Anourosorex yamashinai * ^▲^	Kuroda, 1935		短尾鼩 (臺灣短尾鼩)	3	FishF1/R1; FishF2/R2	NMNS	18, 20, 87	Species endemic to Taiwan
3		Soricidae	Chimarrogale leander*	Thomas, 1902	*Chimarrogale himalayica*	水鼩	3	LepF1/R1	NMNS	47, 82 (2)	
4		Soricidae	Chodsigoa sodalis*	Thomas, 1913		細尾長尾鼩	2	VF2/VR1; FishF1/R1	NMNS	56 (2)	Species endemic to Taiwan
5		Soricidae	*Crocidura shantungensis hosletti*	Jameson and Jones, 1977		荷氏小麝鼩	3	FishF1/R1	NMNS	69 (3)	Taiwanese endemic subspecies
6		Soricidae	Crocidura tadae*	Tokuda and Kano, 1936	*Crocidura rapax tadae*	長尾麝鼩	3	LepF1/R1	NMNS	54 (2), 87	Species endemic to Taiwan
7		Soricidae	*Crocidura tanakae*	Kuroda, 1938		臺灣灰麝鼩	4	LepF1/R1	NMNS	11, 68, 89, 101	
8		Soricidae	*Pseudosoriculus fumidus*	(Thomas, 1913)	*Episoriculus fumidus*	臺灣長尾鼩 (臺灣煙尖鼠)	3	FishF1/R1	NMNS	19, 75 (2)	Species endemic to Taiwan
9		Soricidae	*Suncus murinus*	(Linnaeus, 1766)		臭鼩	3	BatL5310/R6036R	NMNS	38, 69, 85	
10		Talpidae	Mogera insularis* ^▲^	(Swinhoe, 1863)		臺灣鼴鼠	3	FishF2/R2; LepF1/R1	NMNS	4, 63, 64	Species endemic to Taiwan
11		Talpidae	Mogera kanoana* ^▲^	Kawada et al. 2007		鹿野氏鼴鼠	3	LepF1/R1	NMNS	14, 37, 61	Species endemic to Taiwan
12	Pholidota	Manidae	*Manis pentadactyla pentadactyla*	Linnaeus, 1758		臺灣穿山甲	5	FishF1/R1	TPZ, NMNS	5, 26, 60, 92, 96	Taiwanese endemic subspecies
13	Carnivora	Felidae	*Prionailurus bengalensis*	(Kerr,1792)		石虎	5	FishF1/R1; LepF1/R1	TPZ, NMNS	59, 60, 83, 84, 93	
14		Herpestidae	Urva urva formosana*	(Bechthold, 1936)	*Herpestes urva formosanus*	臺灣食蟹獴	5	LepF1/R1, FF2d/FR1d	TPZ, NMNS	12, 13, 32, 60 (2)	Taiwanese endemic subspecies
15		Mustelidae	*Lutra lutra*	(Linnaeus, 1758)		歐亞水獺	3	FF2d/FR1d	NMNS	101 (3)	
16		Mustelidae	*Martes flavigula chrysospila*	Swinhoe, 1866		黃喉貂	3	LepF1/R1	NMNS	26, 40, 58	Taiwanese endemic subspecies
17		Mustelidae	*Melogale subaurantiaca*	(Swinhoe, 1863)	*Melogale moschata subaurantiaca*	臺灣鼬獾	5	FF2d/FR1d	TPZ, NMNS	8, 60, 67 (2), 94	Species endemic to Taiwan
18	Carnivora	Mustelidae	*Mustela nivalis formosana*	Lin et al. 2010		高山小黃鼠狼	3	FF2d/FR1d; FishF2/R2	NMNS	21, 23, 88	Taiwanese endemic subspecies
19		Mustelidae	*Mustela sibirica taivana*	Thomas, 1913		臺灣黃鼠狼	3	FF2d/FR1d; FishF1/R1	NMNS	17, 22, 79	Taiwanese endemic subspecies
20		Ursidae	*Ursus thibetanus formosanus*	Swinhoe, 1864		臺灣黑熊	5	FishF1/R1; FishF2/R2; LCO1490/HCO2198	TPZ, NMNS	29, 30, 31, 33, 41	Taiwanese endemic subspecies
21		Viverridae	*Paguma larvata*	(C. H. Smith, 1827)		白鼻心	3	FF2d/FR1d; FishF2/R2	TPZ, NMNS	1 (2), 78	
22		Viverridae	*Viverricula indica*	(É. Geoffroy Saint-Hilaire, 1803)		麝香貓	3	FF2d/FR1d	TPZ, NMNS	12 (2), 60	
23	Artiodactyla	Bovidae	*Capricornis swinhoei*	Gray, 1862		臺灣野山羊	5	FishF1/R1; FishF2/R2	TPZ	5, 9, 35 (2), 44	Species endemic to Taiwan
24		Cervidae	*Cervus nippon taiouanus*	Blyth, 1860		臺灣梅花鹿	2	FishF2/R2; LCO1490/HCO2198	TPZ	3 (2)	Taiwanese endemic subspecies
25		Cervidae	*Muntiacus reevesi micrurus*	(Sclater, 1875)		臺灣山羌	3	FishF1/R1	NMNS	15, 36, 79	Taiwanese endemic subspecies
26		Cervidae	*Rusa unicolor swinhoii*	(Sclater, 1862)		臺灣水鹿	3	FishF1/R1; FishF2/R2	TPZ, NMNS	6, 34, 43	Taiwanese endemic subspecies
27		Suidae	*Sus scrofa taivanus*	(Swinhoe, 1863)		臺灣野豬	3	FishF2/R2	TPZ	3 (3)	Taiwanese endemic subspecies
28	Rodentia	Cricetidae	Alexandromys kikuchii*	(Kuroda, 1920)	*Microtus kikuchii*	臺灣高山田鼠/臺灣田鼠	3	FishF2/R2	NMNS	19 (2), 55	Species endemic to Taiwan
29		Cricetidae	*Eothenomys colurnus*	(Thomas, 1911)	*Eothenomys melanogaster*	黑腹絨鼠	3	FF2d/FR1d	NMNS	77, 81, 87	
30		Muridae	*Apodemus agrarius*	(Pallas, 1771)		赤背條鼠	3	FishF1/R1; FishF2/R2	NMNS	28, 70, 93	
31		Muridae	*Apodemus semotus*	Thomas, 1908		臺灣森鼠	3	FishF1/R1; FishF2/R2	NMNS	21, 48, 90	Species endemic to Taiwan
32		Muridae	*Bandicota indica*	(Bechstein, 1800)		鬼鼠	3	FishF2/R2	NMNS	51, 65, 95	
33		Muridae	*Micromys minutus*	(Pallas, 1771)		巢鼠	3	FishF2/R2	NMNS	73, 59, 87	
34		Muridae	*Mus formosanus*	Kuroda, 1925	*Mus caroli*	田鼷鼠	3	LepF1/R1	NMNS	39, 52, 71	Species endemic to Taiwan
35		Muridae	*Mus musculus*	Linnaeus, 1758		家鼷鼠	5	LepF1/R1	NMNS	98, 52, 100, 101	
36		Muridae	Niviventer coninga*	(Swinhoe, 1864)		刺鼠	3	LepF1/R1	NMNS	7, 57, 66	Species endemic to Taiwan
37	Rodentia	Muridae	Niviventer culturatus*	(Thomas, 1917)		高山白腹鼠	3	LepF1/R1	NMNS	16, 50, 80	Species endemic to Taiwan
38		Muridae	*Rattus exulans*	(Peale, 1849)		緬甸小鼠	3	FishF2/R2	NMNS	26 (2), 27	Invasive species
39		Muridae	*Rattus losea*	(Swinhoe, 1871)		小黃腹鼠	3	FishF2/R2; BatL5310/R6036R	NMNS	13, 52, 70	
40		Muridae	*Rattus norvegicus*	(Berkenhout, 1769)		溝鼠	3	FishF2/R2	NMNS	38, 65, 68	
41		Muridae	*Rattus tanezumi*	(Temminck, 1844)		亞洲家鼠	3	FishF2/R2	NMNS	38, 68 (2)	
42		Sciuridae	Belomys pearsonii kaleensis* ^▲^	(Swinhoe, 1863)		臺灣小鼯鼠	2	FishF2/R2; BatL5310/R6036R	NMNS	10, 53	Taiwanese endemic subspecies
43		Sciuridae	*Callosciurus erythraeus thaiwanensis*	(Bonhote, 1901)		臺灣赤腹松鼠	3	LepF1/R1	NMNS	2, 72, 99	Taiwanese endemic subspecies
44		Sciuridae	Dremomys pernyi owstoni*	(Thomas, 1908)		荷氏松鼠	3	FishF1/R1	NMNS	24, 49, 76	Taiwanese endemic subspecies
45		Sciuridae	Petaurista grandis* ^▲^	(Swinhoe, 1863)	*Petaurista philippensis grandis*	大赤鼯鼠	4	FishF2/R2; LepF1/R1	NMNS	46, 62 (2), 97	Species endemic to Taiwan
46		Sciuridae	Petaurista lena*	Thomas, 1907	*Petaurista alborufus lena*	白面鼯鼠	3	LepF1/R1	NMNS	45, 47, 74	Species endemic to Taiwan
47		Sciuridae	Tamiops maritimus formosanus*	(Bonhote, 1900)		臺灣條紋松鼠	2	FF2d/FR1d	NMNS	49 (2)	Taiwanese endemic subspecies
48	Lagomorpha	Leporidae	*Lepus sinensis formosus*	Thomas, 1908		臺灣野兔	3	FishF1/R1	NMNS	45, 86, 91	Taiwanese endemic subspecies

^▲^ Non-monophyletic sequences * Novel species sequences in the BOLD database

### DNA extraction and COI amplification

Genomic DNA was extracted using the DNeasy Tissue Kit (QIAGEN) following the standard protocol described by Liu et al. (2024). Briefly, samples were initially suspended in 180 μl of ATL buffer and 20 μl of proteinase K and incubated overnight at 56 °C. During the initial 2 h of incubation, the samples were agitated every 20 min. Following overnight incubation, 200 μl of AL buffer was added to each sample, which was briefly vortexed and incubated for an additional 10 min at 56 °C. Thereafter, 200 μl of 95% ethanol (EtOH) was added, and the mixture was vortexed and centrifuged at 8000 × g for 1 min. The supernatant was then transferred to a DNeasy spin column. The column was washed sequentially with 500 μl of AW1 buffer (centrifugation at 8000 × g for 1 min) and 500 μl of AW2 buffer (centrifugation at 13,500 × g for 4 min). Finally, 200 μl of AE buffer was applied to the spin column membrane and incubated for 10 min at room temperature, followed by centrifugation at 8000 × g for 1 min to elute the purified DNA into a clean microcentrifuge tube.

Each extracted DNA sample was amplified by polymerase chain reaction (PCR) using the universal primers listed in Table 2. We modified the PCR protocol of Jang-Liaw et al. (2023), which consisted of an initial denaturation step at 94 °C for 5 min, followed by 35 cycles of denaturation at 94 °C for 50 s, annealing at 50 °C for 50 s, extension at 72 °C for 1 min, and a final extension step at 72 °C for 5 min. Seven primer pairs were selected and successfully used in this study (Table 2). PCR products were verified by 1.5% agarose gel electrophoresis, purified using ExoSAP-It (USB Corp., Cleveland, OH, USA), and bidirectionally Sanger-sequenced on an Applied Biosystems 3730XL DNA Analyser using the same primers for PCR and the BigDye Terminator Cycle Sequencing FS Ready Reaction Kit v. 3.1 (Applied Biosystems), and the sequences were aligned using MegAlign v. 4.0 (DNA Star Inc.) and by eye using the complementary strand for verification.

**Table 2.** Primer sets used for COI amplification and sequencing of the 157 newly generated barcode sequences in this study. The table lists primer names, primer sequences, source references, and amplification/sequencing outcomes across sampled taxa. Success counts and rates are summarized to indicate primer pairs with higher empirical utility for Taiwanese non-volant terrestrial mammal samples in this study.

**Primer name**	**Sequence (5'-3')**	**Direction**	**No. of samples/species successfully amplified**	**Reference**
Batl5310	CCTACTCRGCCATTTTACCTATG	F	7/3	Robins et al. (2007)
R6036R	ACTTCTGGGTGTCCAAAGAATCA	R		Robins et al. (2007)
FF2d	TTCTCCACCAACCACAARGAYATYGG	F	24/9	Ivanova et al. (2007)
FR1d	CACCTCAGGGTGTCCGAARAAYCARAA	R		Ivanova et al. (2007)
FishF1	TCAACCAACCACAAAGACATTGGCAC	F	37/16	Ward et al. (2005)
FishR1	TAGACTTCTGGGTGGCCAAAGAATCA	R		Ward et al. (2005)
FishF2	TCGACTAATCATAAAGATATCGGCAC	F	41/20	Ward et al. (2005)
FishR2	ACTTCAGGGTGACCGAAGAATCAGAA	R		Ward et al. (2005)
LCO1490	GGTCAACAAATCATAAAGATATTGG	F	3/2	Folmer et al. (1994)
HCO2198	TAAACTTCAGGGTGACCAAAAAATCA	R		Folmer et al. (1994)
LepF1	ATTCAACCAATCATAAAGATAT	F	49/16	Hebert et al. (2004)
LepR1	TAAACTTCTGGATGTCCAAAAA	R		Hebert et al. (2004)
VF2	CAACCAACCACAAAGACATTGGCAC	F	1/1	Untailed VF2_t1 in Ivanova et al. (2007)
VR1 (= FishR1)	TAGACTTCTGGGTGGCCAAAGAATCA	R		Ivanova et al. (2006)

### Data analysis

BOLD is a designated repository for global DNA barcode data. All sequences generated in this study were uploaded to BOLD to facilitate comparisons and identification by other researchers in the future. All barcode sequences were first queried against the GenBank database using the online alignment tool BLAST (Altschul et al. 1990) to confirm the accuracy of specimen identification, although COI fragments for some species were not previously available in GenBank (e.g., *Mogera kanoana*). For clarity, two datasets were used in this study. The first was the original BOLD project dataset, which consisted of 157 newly generated COI sequences produced in this study. This dataset was used to establish the BOLD reference library and to generate BOLD-based project summaries, including distance distributions, cluster sequence analysis, and Barcode Index Number (BIN) assignment results. The second was an expanded Taiwan dataset, which combined the 157 newly generated sequences with 75 additional public COI sequences of Taiwanese non-volant terrestrial mammals retrieved from GenBank (Sayers et al. 2025). Because the aim of this study was to construct and evaluate a Taiwan-focused COI barcode reference library, only public sequences derived from Taiwanese samples were incorporated into the expanded dataset. Public records from non-Taiwanese localities were not included, because they may reflect different taxonomic assignments, geographically structured variation outside Taiwan, and highly uneven sampling among taxa and regions. All additional public Taiwanese sequences retained for analysis were ultimately obtained from GenBank; records encountered through other databases or literature searches were used only when a verifiable GenBank accession was available. Accession numbers, taxonomic names used in this study, original taxon names in the source database, and source references when available are provided in Suppl. material 2.

Public sequences were searched using current species names and relevant synonymous or historical names, and only sequences assignable to Taiwan or Taiwanese endemic taxa were retained. Records were excluded when they were derived from non-Taiwanese localities, represented non-COI mitochondrial regions, showed ambiguous taxonomic assignment, or did not sufficiently overlap the barcode region used for comparison. Taxonomic names of public sequences were updated to follow the current nomenclature of Taiwanese mammals adopted in this study, while original database names were retained in Suppl. material 2 for traceability.

BOLD-based analyses were conducted using the original BOLD project dataset of 157 newly generated sequences. Following Raupach et al. (2018), the analytical tools in the BOLD workbench were used to summarize project-level distance patterns and clustering behavior. Distance Summary analysis was conducted at multiple taxonomic levels, including within species, genus, and family, based on pairwise sequence comparisons. For each taxonomic level, the minimum, mean, maximum, and standard error (SE) of the genetic distances were calculated. Barcode Gap Analysis compared the maximum and mean intraspecific distances with the nearest-neighbor distances to evaluate the presence of a barcode gap and examine distance-based clustering behavior. Distance Distribution Histograms were used to compare the overall distributions of mean intraspecific distances and nearest-neighbor distances as supporting evidence of barcoding performance. All BOLD analyses were performed using the default BOLD settings for COI-5P sequences under the Kimura 2-parameter (K2P) model (Kimura 1980). Sequences containing stop codons were retained in these BOLD summaries so that their effects on distance-based and clustering-based results could be evaluated.

All barcode sequences were subjected to the Barcode Index Number (BIN) system implemented in BOLD, which clusters DNA barcodes to generate operational taxonomic units that closely correspond to species. It should be noted that this analysis is not intended to be phylogenetic; rather, the topology represents a graphical visualization of DNA barcode divergences and putative species clusters (Raupach et al. 2018). BIN analysis was used to examine the overall concordance between molecular clustering and morphological identification and to summarize the proportions of concordant BINs, singleton BINs, and sequences not assigned to BINs. In addition, cluster sequence analysis was conducted in BOLD to group sequences into operational taxonomic units (OTUs) based solely on genetic distance and independent of taxonomic assignment. This analysis was used to examine clustering patterns within and among species and to assess the extent of the intraspecific genetic structure under the applied clustering criteria. For each OTU, the number of sequences assigned to that cluster (count), as well as the average and maximum intra-cluster distances, were recorded. To address the concern that low sample numbers per species could affect estimates of intraspecific variation and barcode gaps, the expanded Taiwan dataset was used for additional phylogenetic and distance-based comparisons. The 157 newly generated sequences and 75 GenBank sequences were aligned and trimmed to a shared 527-bp COI region to maximize overlap among sequences. The expanded alignment contained 232 sequences and was analyzed using MEGA 12.1 (Kumar et al. 2024). Neighbor-Joining (NJ) trees were constructed using the K2P model (Kimura 1980; Saitou and Nei 1987). The resulting tree was used to visualize the placement of newly generated sequences relative to public Taiwanese sequences and to identify sequences or taxa showing anomalous clustering patterns.

Species-level barcode-gap comparisons were calculated for both the original and expanded Taiwan datasets. Pairwise K2P distance matrices were generated in MEGA 12.1, and species-level summaries were calculated from the resulting distance matrices in Microsoft Excel. For each species, the maximum intraspecific and nearest-neighbor distances were recorded. The barcode gap was defined as the difference between the nearest-neighbor distance and the maximum intraspecific distance. Species with nearest-neighbor distances greater than maximum intraspecific distances were treated as having positive barcode gaps, whereas species with the maximum intraspecific distances equal to or greater than the nearest-neighbor distances were highlighted for further inspection. These comparisons were used to evaluate whether the addition of public GenBank sequences altered the barcode-gap patterns observed in the original BOLD project dataset.

Potential numts or COI-like pseudogene sequences were screened using multiple lines of evidence to confirm their authenticity. All newly generated and public sequences in the trimmed alignment were translated into amino acid sequences to detect stop codons or reading-frame interruptions. Sequences were further flagged when they showed anomalous NJ placement, unusually high intraspecific divergence, negative barcode-gap context, or inconsistency between full-length sequence inspection and the trimmed alignment. These flags were treated as sequence-quality and numt-screening metadata rather than direct taxonomic evidence. A summary of the suspected numts, stop-codon-containing sequences, and putative COI-like pseudogene sequences is provided in Suppl. material 4, and the complete NJ tree for the expanded Taiwan dataset is provided in Suppl. material 5.

## Results

### Barcode library and sampling coverage

**Figure 2.**
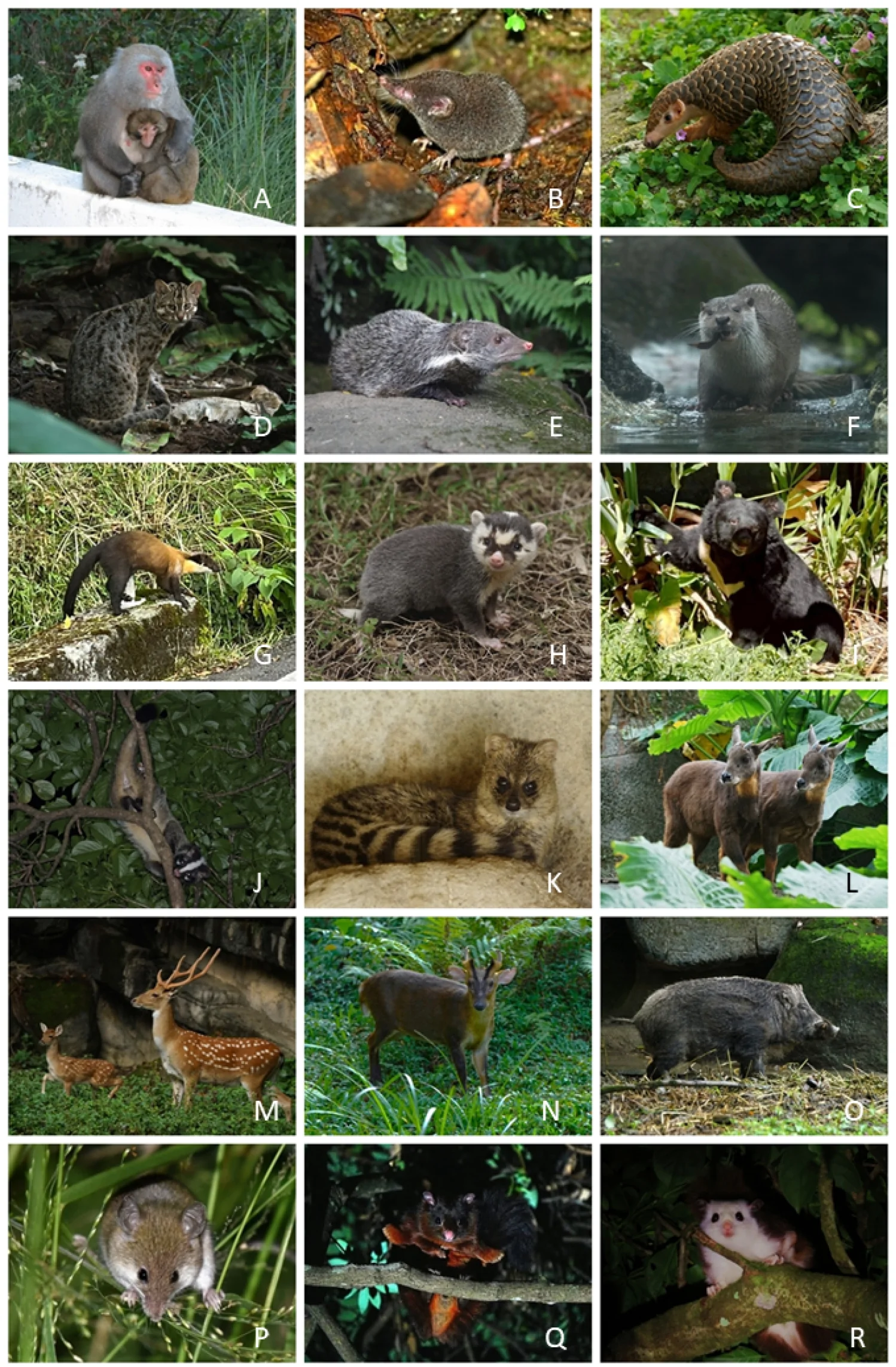
Photographs of selected Taiwanese non-volant terrestrial mammal species included in the barcode library. Numbers in parentheses correspond to species numbers in Table 1. **A**. *Macaca cyclopis* (1), wild individuals; **B**. *Crocidura tadae* (6), wild individual; **C**. *Manis pentadactyla pentadactyla* (12), rescued individual at Taipei Zoo; **D**. *Prionailurus bengalensis* (13), rescued individual; **E**. *Urva urva formosana* (14), rescued individual; **F**. *Lutra lutra* (15), captive individual at Taipei Zoo; **G**. *Martes flavigula chrysospila* (16), wild individual; **H**. *Melogale subaurantiaca* (17), rescued individual; **I**. *Ursus thibetanus formosanus* (20), rescued individual; **J**. *Paguma larvata* (21), wild individual; **K**. *Viverricula indica* (22), rescued individual; **L**. *Capricornis swinhoei* (23), captive individual; **M**. *Cervus nippon taiouanus* (24), captive individual; **N**. *Muntiacus reevesi micrurus* (25), captive individual; **O**. *Sus scrofa taivanus* (26), captive individual; **P**. *Mus formosanus* (34), wild individual; **Q**. *Petaurista grandis* (45), wild individual; **R**. *Petaurista lena* (46), wild individual. Photo credits: A, Shih-Wei Chang; B, P, Q, Chi-Ho Tsai; C–F, H, J–O, R, Te-Chuan Chan; G, Nian-Hong Jang-Liaw; I, Chia-Chieh Chien.

A total of 157 newly generated COI DNA barcode sequences were generated. The sequence lengths ranged from 581 to 724 bp, with a mean of 682 bp. The sequences reported here represent 48 recognized species from seven orders, 16 families, and 37 genera, including the vast majority of endemic and common extant non-chiropteran terrestrial mammals currently recorded on the island of Taiwan (Table 1; Suppl. material 1). The sampling localities of the newly generated sequences covered the main island of Taiwan and offshore island localities represented in this study, including Kinmen for *Lutra lutra* (Fig. 1). Photographs of the selected Taiwanese mammal species included in the barcode library are presented in Fig. 2. For 15 of the 48 sampled species/subspecies (31.25%), no COI barcode record was available in BOLD prior to this study (Table 1). Accordingly, within the sampled taxon set, BOLD coverage increased from 33/48 taxa (68.75%) before this study to 48/48 taxa (100%) after deposition, and these 15 taxa received their first BOLDCOI barcode records through this project. All 157 sequences were linked to voucher or tissue-archive information from the NMNS or TPZ, following strict data traceability standards, and uploaded to the BOLD workbench under the project “Terrestrial Mammals of Taiwan (except Chiroptera); TPZMM”.

### Primer performance

Table 2 summarizes the probabilities of successful PCR amplification for each primer pair in this study. Different species showed differential primer suitability (Table 1). LepF1/R1 yielded the largest number of successfully sequenced samples, amplifying 49 samples representing 16 species, whereas FishF2/R2 covered the largest number of species, amplifying 41 samples representing 20 species. FishF1/R1 also showed broad applicability, amplifying 37 samples representing 16 species. Together, these three primer pairs successfully amplified 80.89% of the samples (127/157), representing 42 species (87.5%). Other primer pairs were useful for a smaller number of taxa or samples, indicating that no single primer pair was universally effective across all non-volant terrestrial mammal taxa examined in this study.

### Expanded Taiwan dataset and NJ tree analysis

To evaluate whether the barcode patterns observed in the original BOLD project dataset were robust to broader sampling, 75 additional public COI sequences of Taiwanese non-volant terrestrial mammals were incorporated from GenBank (Suppl. material 2). The expanded Taiwan dataset therefore included 232 sequences, comprising the 157 newly generated sequences and 75 public sequences. Public sequences were available for 35 of the 48 taxa, whereas no additional public COI sequence was available for the remaining 13 taxa.

**Figure 3.**
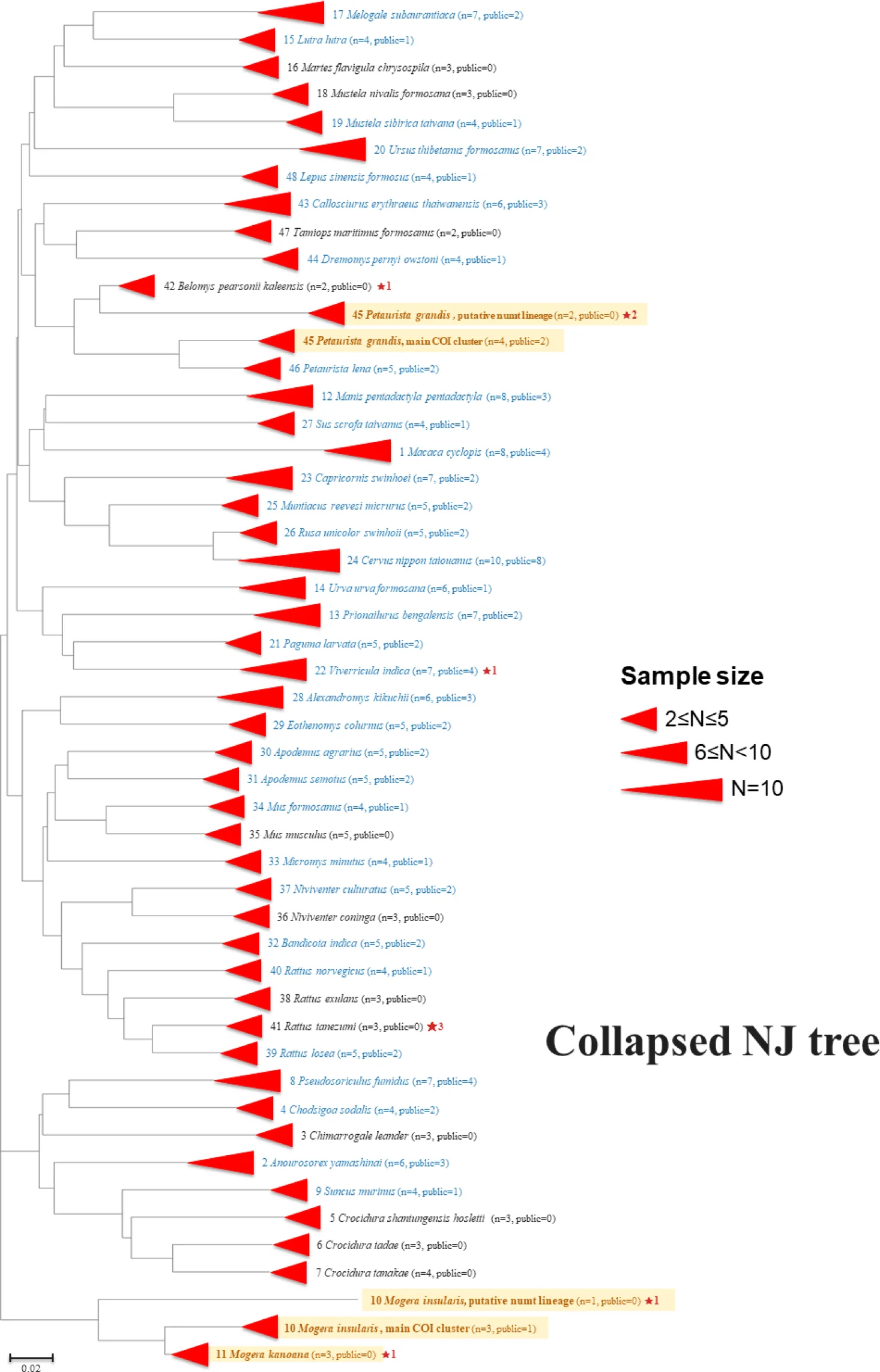
Collapsed Neighbor-Joining (NJ) tree of the expanded Taiwan COI dataset, including 157 newly generated sequences and 75 public GenBank sequences of Taiwanese non-volant terrestrial mammals. The tree was constructed from the 527-bp trimmed COI alignment using the Kimura 2-parameter (K2P) model. Red triangles indicate collapsed species-level or lineage-level clusters, with triangle size corresponding to the number of sequences included in each cluster. Species names are preceded by species numbers corresponding to Table 1. Values in parentheses indicate the number of newly generated sequences and public GenBank sequences included for each taxon or lineage. Blue labels indicate taxa for which public GenBank sequences were included in the expanded dataset. Red stars denote stop-codon-containing sequences and/or putative numts, with the number indicating the number of flagged sequences. Yellow highlighting indicates lineages requiring further inspection, including the main COI clusters and putative numt lineages of *Mogera insularis* and *Petaurista grandis*, and the flagged *Mogera kanoana* lineage. The scale bar represents a 0.02 K2P distance.

The NJ tree based on the 527-bp trimmed alignment of the expanded Taiwan dataset showed that most newly generated sequences clustered with conspecific public sequences or with other newly generated conspecific sequences (Fig. 3; Suppl. material 5). This result generally supports the species-level identification performance of COI barcodes for most non-volant terrestrial mammals in Taiwan. However, several sequences or taxa showed anomalous placements or split clustering patterns. These included *Mogera insularis*, *M. kanoana*, *Petaurista grandis*, and *Rattus tanezumi*, which were therefore highlighted for further inspection (Fig. 3; Suppl. material 4).

### Barcode-gap comparison between the original and expanded datasets

**Figure 4.**
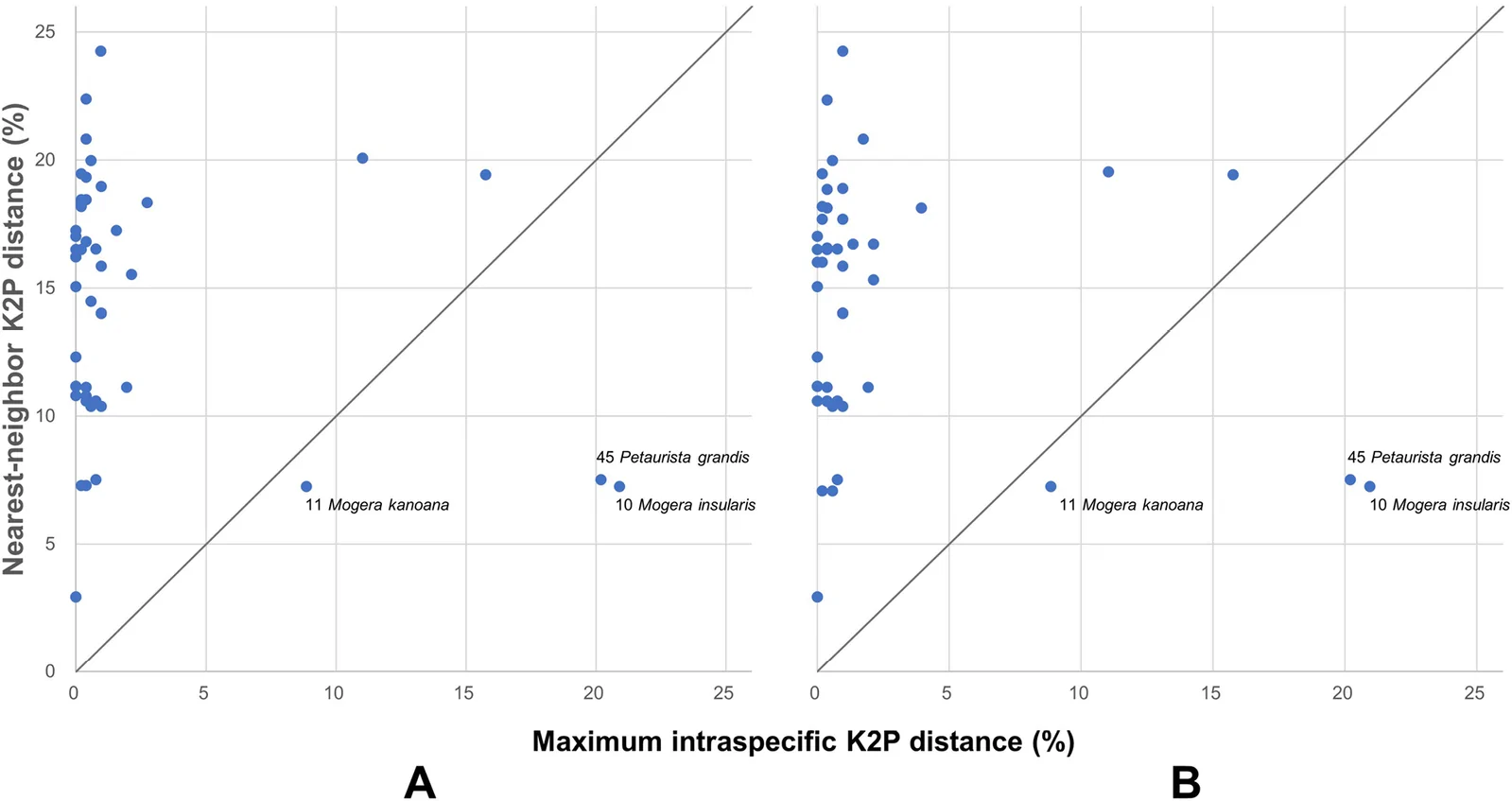
Barcode-gap comparison based on species-level K2P distances in the original and expanded Taiwan COI datasets. **A**. Original dataset including 157 newly generated COI sequences; **B**. Expanded Taiwan dataset including 157 newly generated sequences and 75 public GenBank sequences. Each point represents one taxon. The x-axis shows the maximum intraspecific K2P distance, and the y-axis shows the nearest-neighbor K2P distance. Points above the diagonal line indicate positive barcode gaps, whereas points below the diagonal line indicate negative barcode gaps. Taxa showing negative barcode gaps are labelled with species numbers corresponding to Table 1: 10, *Mogera insularis*; 11, *Mogera kanoana*; and 45, *Petaurista grandis*. Species-level distance values and barcode-gap summaries are provided in Suppl. material 3.

Species-level barcode-gap comparisons showed that the addition of public GenBank sequences did not substantially alter the overall barcode-gap pattern. In both the original dataset and the expanded Taiwan dataset, 45 of the 48 taxa retained positive barcode gaps, indicating that nearest-neighbor distances were greater than maximum intraspecific distances for most taxa (Fig. 4; Suppl. material 3). Three taxa showed negative barcode gaps in both datasets: *Mogera insularis*, *M. kanoana*, and *Petaurista grandis* (Fig. 4; Suppl. material 3). In *M. insularis*, the maximum intraspecific K2P distance was 20.89% in the original dataset and 20.94% in the expanded dataset, whereas the nearest-neighbor distance to *M. kanoana* was 7.26%, resulting in negative barcode gaps of – 13.63% and – 13.68%, respectively. In *M. kanoana*, the maximum intraspecific distance was 8.86%, whereas the nearest-neighbor distance to *M. insularis* was 7.26%, resulting in a negative barcode gap of – 1.60% in both datasets. In *P. grandis*, the maximum intraspecific distance was 20.19%, whereas the nearest-neighbor distance to *Petaurista lena* was 7.52%, resulting in a negative barcode gap of – 12.67% in both datasets.

These three taxa corresponded to the major cases of split clustering or anomalous sequence placement in the expanded NJ tree (Fig. 3; Suppl. material 5). In contrast, several taxa containing flagged sequences, including *Viverricula indica*, *Belomys pearsonii kaleensis*, and *Rattus tanezumi*, retained positive barcode gaps at the species level, suggesting that barcode-gap status alone was insufficient to detect all putative sequence artefacts or numt-related problems.

### Suspicious sequences and putative numts

In the standardized 527-bp trimmed alignment, six newly generated sequences from five taxa contained stop codons and were flagged as stop-codon-containing sequences or putative numts (Suppl. material 4). These included one sequence of *Mogera insularis*, one sequence of *M. kanoana*, one sequence of *Viverricula indica*, one sequence of *Belomys pearsonii kaleensis*, and two sequences of *Petaurista grandis*. These sequences showed variable degrees of supporting evidence, including stop codons, anomalous NJ placement, negative barcode-gap context, or split clustering patterns.

The three newly generated *Rattus tanezumi* sequences were also flagged as putative numts based on the revised NJ tree and full-length sequence inspection, although they were not scored as stop-codon-containing sequences in the standardized 527-bp trimmed-alignment screen (Suppl. material 4). This made *R. tanezumi* a notable case in which all newly generated sequences for a species were treated as putative numts, even though the species retained a positive barcode gap in the distance-based comparison. Thus, the *R. tanezumi* sequences should be interpreted cautiously in the reference library until authentic mitochondrial COI sequences are confirmed for Taiwanese samples.

However, a small number of species exhibited comparatively high levels of intraspecific divergence, which in some cases exceeded interspecific distances. The most prominent examples were *M. insularis* and *P. grandis*, both of which showed maximum intraspecific distances exceeding 20% and negative barcode gaps in both the original and expanded datasets (Suppl. material 3). Interestingly, for the *M. insularis* sample T0019115 (Process ID TPZMM001-21), in addition to successful amplification and sequencing using FishF2/R2, we also obtained PCR amplification using LepF1/R1; however, the sequencing quality was poorer, and only a reverse-direction sequence of approximately 351 bp (primed by LepR1) was obtained. This trace file was archived under the “Supporting Traces” option on the BOLD Sequence Page for this sample. We ultimately discarded the LepF1/R1 sequencing result and deposited the 676-bp bidirectional sequence generated using FishF2/R2 in the BOLD database. Notably, the discarded 351-bp sequence, when compared with the 676-bp sequence from the same DNA sample, showed an unexpectedly large divergence; instead, it was more similar to two other *M. insularis* samples (NMNS018817 and NMNS019226) that formed a different cluster in the NJ tree, with sequence similarities of 98.86% and 98.01%, respectively. The two samples were amplified and bidirectionally sequenced using the LepF1/R1 primers. This case indicates that different primer pairs may amplify divergent COI-like fragments from the same DNA sample and highlights the need to interpret anomalous barcode sequences together with trace quality, translation results, tree placement, and barcode-gap context.

### BOLD-based distance distribution and BIN clustering results

**Figure 5.**
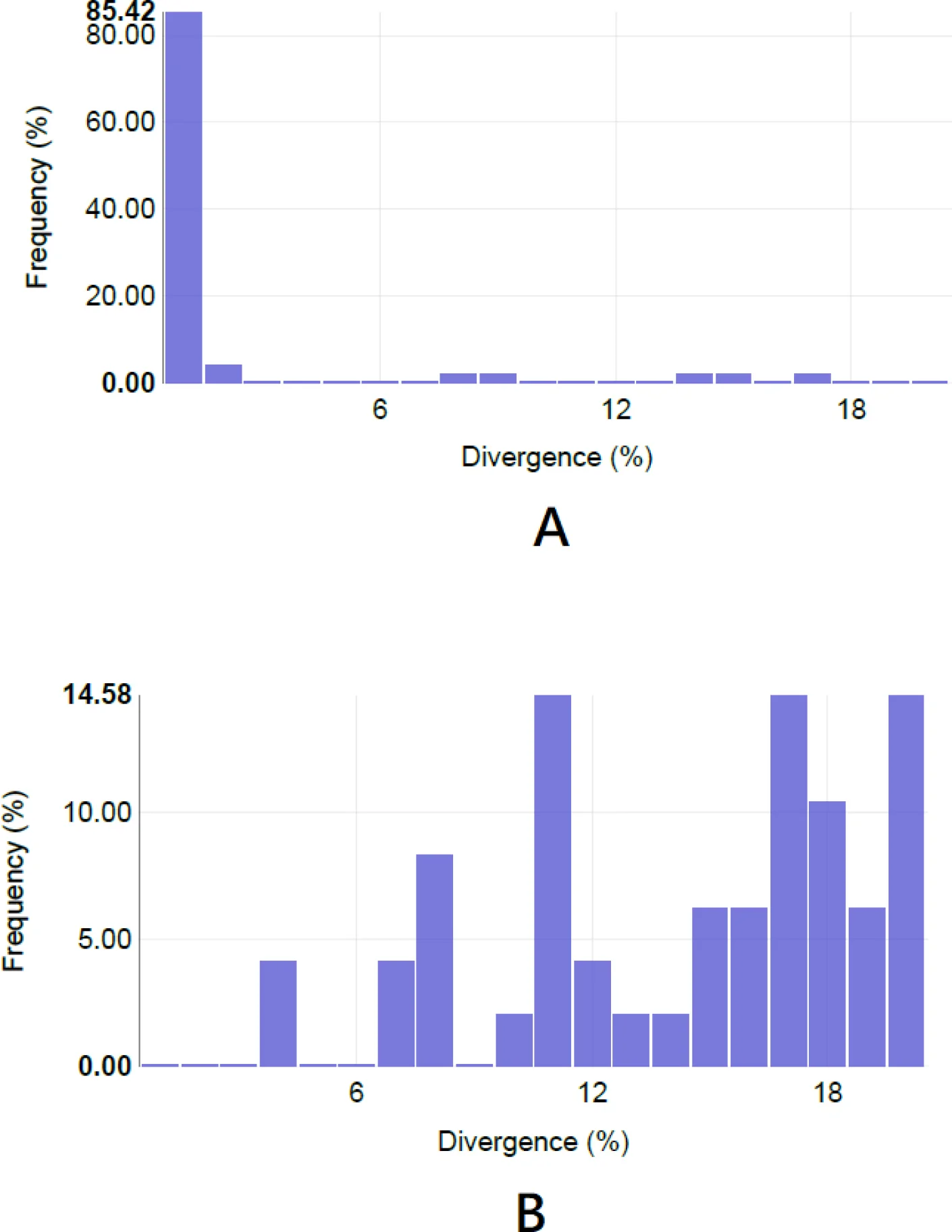
Distance-distribution histograms of COI sequence divergence generated from the original BOLD project dataset of 157 newly generated sequences. **A**. Distribution of mean intraspecific K2P distances among the 48 sampled taxa; **B**. Distribution of nearest-neighbor K2P distances among the sampled taxa. These histograms provide a BOLD-based project-level visualization of the separation between within-taxon divergence and nearest-neighbor divergence in the newly generated barcode library.

**Figure 6.**
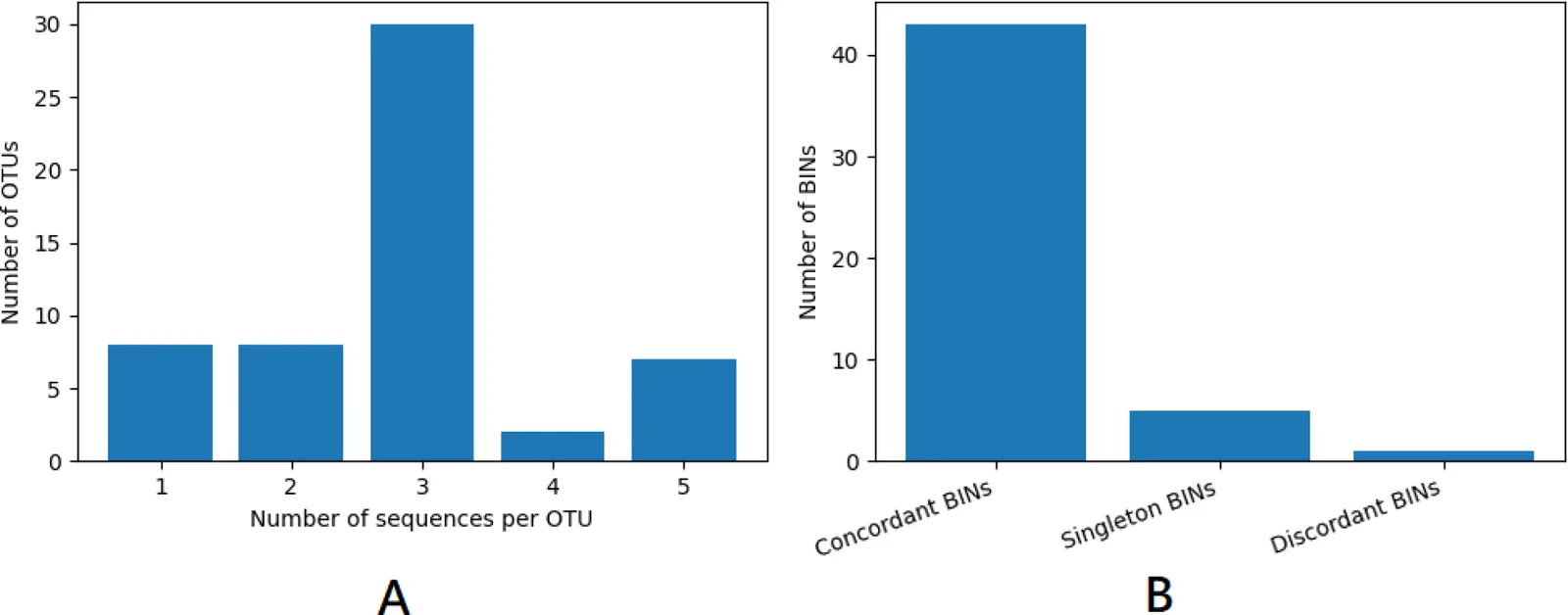
BOLD-based clustering and Barcode Index Number (BIN) assignment summaries for the original BOLD project dataset of 157 newly generated COI sequences. **A**. Sequence clustering analysis grouped the COI sequences into 55 operational taxonomic units (OTUs), with each OTU containing one to five sequences; **B**. BIN discordance analysis categorized assigned BINs as concordant, singleton, or discordant. Concordant BINs contain sequences assigned to a single taxon, singleton BINs are represented by a single sequence, and discordant BINs include sequences assigned to more than one taxon. These BOLD-based summaries should be interpreted as clustering and BIN-assignment results for the newly generated reference-library sequences, rather than for the expanded Taiwan dataset that includes public GenBank sequences.

BOLD-based project summaries were generated using the original BOLD project dataset with 157 newly generated sequences. The Distance Distribution Histograms showed that mean intraspecific distances were concentrated at low divergence values, whereas nearest-neighbor distances were distributed mainly at higher values, indicating a general separation between within-taxon and nearest-neighbor divergence in the original dataset (Fig. 5). This pattern was consistent with the barcode-gap comparison and supported the discriminatory power of COI barcodes for most sampled taxa. Cluster sequence analysis grouped the 157 COI sequences into 55 operational taxonomic units (OTUs), with each OTU containing between one and five sequences (Fig. 6A). The BIN Discordance analysis identified 43 concordant BINs, five singleton BINs, and one discordant BIN (Fig. 6B). A total of 145 of the 157 newly generated sequences were assigned to 49 BINs, whereas 12 sequences were not assigned to BINs. The discordant BIN was treated as a flag for further inspection rather than as direct taxonomic evidence.

Taken together, the expanded NJ tree, barcode-gap comparison, Distance Distribution Histograms, Cluster Sequence Analysis, and BIN results indicate that most species exhibited low intraspecific genetic distances, clear barcode gaps, and formed single or highly consistent molecular clusters. However, a small number of species showed higher intraspecific divergence, negative barcode gaps, split clustering patterns, or putative numt-related sequence artefacts. These cases were concentrated in *M. insularis*, *M. kanoana*, *P. grandis*, and *R. tanezumi*, with additional stop-codon-containing sequences detected in *V. indica* and *B. pearsonii kaleensis*. If not recognized, such sequences may lead to overestimation of intra-specific distances and interfere with the interpretation of BIN clustering and barcode gaps.

## Discussion

### Coverage, curation, and broader value of the Taiwan mammalian COI barcode library

Although the 157 newly generated sequences in this study are numerically modest compared with some recent large-scale barcoding campaigns, sequence count alone does not capture the contribution of regional reference libraries. Of the 48 taxa sampled here, 15 (31.25%) lacked any COI barcode record in the BOLD before this study; their addition increased BOLD representation of the sampled taxon set from 33/48 taxa (68.75%) to 48/48 taxa (100%). Accordingly, a major contribution of this project is not merely the addition of sequence volume, but the closure of complete taxonomic reference gaps for Taiwanese mammals, including species and subspecies that were previously unrepresented in BOLD. The remaining limitation is within-taxon depth: several species are represented by few individuals; therefore, the present library should be regarded as a taxonomically broad baseline that can be progressively expanded with additional geographic and population-level sampling.

The value of these records is further strengthened by their provenance. Each newly generated barcode is linked to a museum voucher or an archived tissue record from the National Museum of Natural Science or Taipei Zoo, together with catalogue and locality information. Such linkages permit subsequent re-examination of specimen identity when taxonomy changes, allow anomalous sequences to be traced back to their source material, and create a reproducible connection between molecular identification and curated biological collections. Reference-library frameworks specifically emphasize traceability as a central requirement for reliable barcode identification (Gwiazdowski 2024). Museum and cryobank collections are particularly valuable for mammals, for which obtaining new voucher material can be logistically or ethically difficult, and archived specimens can fill taxonomic gaps that would otherwise persist (Schäffer et al. 2017).

These features provide the library with utility beyond the current identification tests. Voucher-validated local references can support taxonomic verification, identification of morphologically ambiguous or degraded material, conservation research, wildlife forensic investigations, and ecological applications in which unknown mammalian DNA is recovered from tissues, hair, scat, prey remains, and other non-invasive samples. Regional mammal barcode datasets have been shown to facilitate biodiversity assessment and conservation-oriented identification (Francis et al. 2010; Jang-Liaw 2021), whereas validated mammalian reference libraries are directly relevant to the forensic identification of threatened and traded species (Wilson et al. 2016; Baxter et al. 2024). Locally curated records are especially useful for endemic lineages and subspecies in Taiwan because geographically distant reference sequences may not adequately represent regional mitochondrial diversity or current local taxonomy.

More broadly, this study illustrates why regional and taxonomically curated barcode libraries remain valuable even when they are not exceptionally large. Large global repositories maximize discoverability and comparative scope; however, their practical identification performance still depends on whether focal taxa are represented by correctly identified and traceable records. Therefore, a smaller library that closes regional taxonomic gaps, documents vouchers and metadata, and explicitly identifies uncertain records can have high scientific utility and serve as a curated anchor for future expansion (Francis et al. 2010; Gwiazdowski 2024). The integration of museum and cryobank resources in the present study also provides an extensible framework in which additional individuals, geographic populations, and newly recognized taxa can be added while retaining continuity with the verified source material.

The high species-level resolution observed in the expanded NJ tree and barcode gap comparison further supports the practical utility of this library. After incorporating 75 additional Taiwanese COI sequences from GenBank, 45 of the 48 taxa retained positive barcode gaps, indicating that the overall conclusion of good COI-based discriminatory power was not substantially changed by the addition of public data. This provides a useful robustness check on the original dataset, but it does not eliminate the limitation of uneven sampling. Public sequences were unevenly distributed among taxa, and several species still had few or no additional Taiwanese COI records. Therefore, the expanded dataset should be viewed as a broader comparative framework rather than a complete assessment of intraspecific variation across all Taiwanese mammal populations.

The occurrence of a small number of singleton and discordant BINs also shows why a curated reference library should preserve uncertainty rather than reduce all records to a single identification score. BIN-based clustering may be influenced by intraspecific structure, sampling density, taxonomic problems, or sequence-related artifacts. In this study, negative barcode gaps, split clustering patterns, and anomalous tree placements were concentrated in a small number of taxa, especially *Mogera insularis*, *M. kanoana*, *Petaurista grandis*, and *Rattus tanezumi*. These cases demonstrate that project-level summary statistics are useful for screening overall performance, but taxon-specific anomalies require individual inspection and, where necessary, explicit annotation in the reference library.

### Primer usage

A simple and rapid workflow is a key element of DNA barcoding. Identifying PCR primers that can be applied to unknown samples from diverse taxa remains a challenge. This study included 48 species from seven orders and 16 families, representing substantial genetic divergence. Currently, no universal primer pair is suitable for all taxa. Therefore, we provide empirically based recommendations for primer pairs that successfully amplify each species (Table 1) in this study. The results indicated that LepF1/R1, FishF2/R2, and FishF1/R1 were the most successful primer pairs; when the taxonomic identity of an unknown sample is uncertain, these three primer pairs should be prioritized for PCR. However, the primer options are limited for some species. For example, in this experiment, all primer pairs listed in Table 2 failed for the shrew, except for Bat/R6032R. If PCR amplification cannot be achieved when the identity of an unknown sample is uncertain, the species identification function of DNA barcoding will be less widely adopted in the future. The primer success probabilities provided in Table 2 can help future researchers prioritize primer selection for mammalian barcoding studies and reduce trial and error. It should be noted that, except for the shrew, we did not test all the primer pairs listed in Table 2 for every sample. The primer pairs listed for each species in Table 1 are those that successfully amplified and sequenced the species in this study. Primer pairs not listed may have failed in that species, but may also simply not have been tested; thus, their absence does not necessarily indicate failure of the primers. Accordingly, the primer pairs listed for each species in Table 1 are recommended as priority choices, whereas unlisted primer pairs may still be considered as candidates but can be ranked after those with demonstrated success.

The primer-related results also have implications for the detection of numts. Different primer pairs may preferentially amplify different mitochondrial or COI-like fragments, particularly when degraded DNA, low-template DNA, or primer-template mismatches are involved. The case of *M. insularis* sample T0019115 is especially informative because two primer pairs generated markedly different sequence information from the same DNA sample. Although the longer FishF2/R2 product was of higher sequencing quality and was deposited in BOLD, the shorter LepF1/R1-derived fragment was more similar to other *M. insularis* sequences. This suggests that primer choice and sequence quality should be evaluated together, and that a technically clean sequence may still require biological interpretation when tree placement, translation results, or barcode-gap context are anomalous.

### Effects of expanded sampling and public GenBank sequences

The addition of 75 public GenBank sequences provided an opportunity to evaluate whether the original BOLD project dataset was affected by limited specimen numbers per species. Overall, the expanded Taiwan dataset supported the main conclusions from the original dataset: most taxa remained clearly separable from their nearest neighbors, and the general pattern of low within-taxon divergence and higher nearest-neighbor divergence was retained. This indicates that the original voucher-linked dataset is suitable as a reference library for most Taiwanese non-volant terrestrial mammals.

Simultaneously, the expanded dataset clarified which taxa require caution. *Mogera insularis*, *M. kanoana*, and *Petaurista grandis* showed negative barcode gaps in both the original and expanded datasets. These results should not be immediately interpreted as evidence of taxonomic invalidity or species synonymy. Instead, they indicate that the current COI dataset contains heterogeneous sequence clusters that may reflect a combination of numts or COI-like pseudogenes, historical taxonomic complexity, geographic structure, primer-related amplification differences, or limited comparative sampling. Additional analyses using independently inherited nuclear markers, broader geographic sampling, and careful re-examination of voucher specimens would be required before drawing taxonomic conclusions from these patterns.

The expanded dataset also illustrates the limitation of relying solely on barcode-gap status. Some taxa containing flagged sequences still retain positive barcode gaps at the species level. Therefore, a positive barcode gap does not necessarily guarantee that all included sequences represent authentic mitochondrial COI. This is particularly important for reference libraries, because even a small number of pseudogene-derived sequences may affect subsequent identifications if they are used uncritically as reference records.

### Numts, COI-like pseudogenes, and interpretation of anomalous sequences

Although consistent with the conclusions from previous mammalian barcoding studies that COI barcodes generally provide good discriminatory power for Taiwan’s non-volant terrestrial mammals, this study also shows that the presence of numts or COI-like pseudogene sequences can substantially interfere with molecular identification. Sequences containing stop codons occupied implausible positions in phylogenetic analyses and produced extreme values in distance analyses; if not detected, they could be misinterpreted as high intraspecific divergence or potential cryptic species. This concern is not only theoretical but also practical. Pseudogene-related problems have already been reported in the Taiwanese DNA-barcoding context (Chen et al. 2024; da Cruz et al. 2019; de Flamingh et al. 2023), and mammalian datasets from other regions have also shown that numts can affect COI barcoding or mitochondrial sequence reconstruction. The present study adds a Taiwanese mammalian case study showing how such sequences can influence BOLD/BIN interpretation, barcode-gap patterns, and NJ tree topology.

In the case of the *Mogera insularis* sample T0019115, two barcode primer pairs generated two markedly different sequences from the same DNA sample, and the sequence generated by the primer pair with lower sequencing quality may better represent the true COI fragment. This suggests that a numt fragment (i.e., the sequence deposited in BOLD for T0019115) may compete with the COI fragment during PCR because of sequence homology and be preferentially amplified and successfully sequenced, thereby masking the authentic COI fragment. A similar interpretive problem was observed in several other flagged sequences, including *M. kanoana*, *Viverricula indica*, *Belomys pearsonii kaleensis*, and *Petaurista grandis*, where stop codons, anomalous tree placement, or barcode-gap context suggested that the sequences should not be treated as ordinary mitochondrial COI records without qualification

*Rattus tanezumi* represents a different but important case. In the revised screening, all three newly generated *R. tanezumi* sequences were flagged as putative numts based on tree placement and full-length sequence inspection, even though the standardized 527-bp trimmed-alignment screen did not score them as stop-codon-containing sequences. This indicates that translation-based screening alone may not detect all problematic COI-like sequences. For *R. tanezumi*, the presence of numerous public COI records in sequence databases suggests that authentic mitochondrial COI sequences are available for comparison. However, the sequences generated in this study should be interpreted cautiously until they are confirmed by additional primers, longer mitochondrial fragments, or genomic evidence.

In DNA barcoding studies, relying solely on distances or tree topology for interpretation can lead to erroneous conclusions, particularly for species with high intraspecific divergence. Because both BIN and Barcode Gap Analysis rely on genetic distances among sequences, the inclusion of numt sequences or COI-like pseudogene sequences may cause incorrect clustering results or misinterpretation of barcode gaps (Bensasson et al. 2001; Song et al. 2008). Therefore, COI-based identification should integrate functional sequence checks, distance distributions, comparisons with public data, and tree structure interpretation. The detection of stop codons and other translation-based checks is important, but the *R. tanezumi* case shows that the absence of stop codons in a trimmed barcode alignment does not completely exclude a putative numt or COI-like pseudogene. Therefore, suspicious sequences should be evaluated using multiple criteria, including chromatogram quality, translation results, full-length sequence inspection, primer history, tree placement, barcode-gap context, and comparison with independent reference sequences.

From a reference-library perspective, the key methodological lesson is that correct specimen identification and sequence authenticity are separate quality-control problems. A barcode derived from a correctly identified voucher can still be an unsuitable mitochondrial reference if a numt is preferentially amplified. Consequently, suspicious records should remain linked to their vouchers, trace files, and primer metadata, but should be explicitly flagged and not treated as validated mitochondrial references until independently confirmed. The T0019115 and *R. tanezumi* cases further show that no single diagnostic criterion—clean chromatograms, absence of stop codons, a positive barcode gap, or BIN assignment—is sufficient on its own to identify a species. Translation checks, full-length inspection, primer history, replicate amplification with alternative primers where feasible, tree placement, distance context, and comparison with independent public references should be used in combination. This multi-evidence curation approach reduces the risk of numts inflating apparent intraspecific diversity, creating artificial clusters, or propagating erroneous identifications in downstream studies (Bensasson et al. 2001; Song et al. 2008; da Cruz et al. 2019; Gwiazdowski 2024).

### Issues related to Formosan black bear samples

Among the four Formosan black bear samples processed in this study, one COI sequence differed markedly from the other three, contributing to a higher intraspecific K2P genetic distance for this taxon. This distinct sample (NMNSC19426) originated from a private farm in southern Taiwan (“King of snake of the world education farm”). Although recorded as a Formosan black bear (the Taiwan subspecies of the Asian black bear, *Ursus thibetanus formosanus*), the COI comparisons in this study indicated that it represented an Asian black bear that did not originate from Taiwan. Similar cases are not uncommon in Taiwan. Over the past ~20 years, many captive bears abandoned or donated to rescue facilities or zoos have been identified through molecular methods as non-native Asian black bears or hybrids rather than the native Formosan subspecies. These likely smuggled non-native bears are difficult to distinguish from native bears based on external morphology and have historically been treated as Formosan black bears. In contrast, truly native Formosan black bears may be relatively rare in captive ex situ populations (Shih 2018). The other three samples analyzed here were residual blood samples collected during health examinations of bears involved in government-authorized rescue, medical treatment, or holding operations conducted with assistance from the Taipei Zoo. These samples were approved by the competent authorities and archived at the Taipei Zoo Cryobank. The source individuals for these three samples were wild bears, and their DNA sequences represent the genetic information of the native Formosan black bear.

Although this case did not produce a negative barcode gap in the revised species-level comparison, it demonstrates an important applied value of barcode reference libraries. Molecular identification can detect inconsistencies between historical labels, captive-origin records, and the genetic identity of sampled individuals. For conservation-sensitive taxa such as the Formosan black bear, voucher-linked and source-verified reference sequences are essential because misidentified captive or non-native individuals may otherwise be incorporated into reference datasets and affect downstream conservation, forensic, or population-level interpretations.

### Discussion and recommendations regarding the taxonomic status of ferret-badgers

Ferret-badgers are the most common mustelids in Taiwan. The IUCN Red List treats ferret-badgers as LC and does not subdivide them into subspecies (Duckworth et al. 2024). Taiwan’s ferret-badger is generally considered a South China subspecies (*Melogale moschata subaurantiaca*). However, the Mammal Diversity Database (MDD), following analyses of mtDNA cyt b fragments by Rozhnov et al. (2019), reverted Taiwan’s ferret-badger to Swinhoe’s (1863) original description and elevated it to species rank (Mammal Diversity Database 2025). Our COI results are consistent with this view, showing >3% divergence from populations in mainland China and Vietnam, exceeding the 2.2% threshold suggested by Ratnasingham and Hebert (2013), and supporting the classification of the Taiwanese population as a valid distinct species. Nevertheless, current discussions on species validity in Taiwanese ferret-badgers are based solely on DNA data, and detailed comparative analyses of skeletal and anatomical morphology among specimens from different regions are lacking. We concur with the recommendations of Hosoda et al. (2011) and Rozhnov et al. (2019) that a detailed and thorough comparative anatomical analysis is required to clarify its taxonomic status. In addition, Rozhnov et al. (2019) included Taiwan in the distribution of the widespread species *M. moschata*, implying that two *Melogale* species occur in Taiwan. Among our five samples, we did not detect sequences that could be attributed to *M. moschata*; thus, we do not support the hypothesis that Taiwan harbors two *Melogale* species. A more comprehensive comparison of the morphology and DNA of the ferret-badger populations in Taiwan is required.

The ferret-badger case also illustrates the appropriate scope of this study. COI barcodes can provide useful evidence for identifying divergent lineages and evaluating whether Taiwanese populations are distinct from nearby continental populations. However, species delimitation should not rely solely on a single mitochondrial marker. Therefore, our COI results support the need for continued taxonomic attention to Taiwanese ferret-badgers but should be integrated with nuclear markers, morphology, and broader geographic sampling before making definitive taxonomic revisions.

## Conclusions

This study established a curated, voucher-linked COI DNA barcode reference library for 48 non-volant terrestrial mammal taxa from Taiwan. Although the dataset contains a modest number of newly generated sequences compared with large-scale barcode campaigns, its contribution is substantial at the level most relevant to a regional reference resource: 15 of the 48 sampled taxa (31.25%) previously lacked COI barcode records in BOLD. Their inclusion increased BOLD coverage within the sampled taxon set from 68.75% to 100% and provided the first BOLDCOI records for several Taiwanese species and subspecies. Because the sequences are linked to museum vouchers or archived tissue records and associated metadata, the library provides an auditable reference that can be re-examined as taxonomic changes and can support future taxonomic, ecological, conservation, wildlife-monitoring, and forensic applications.

An expanded comparison with 75 public Taiwanese COI sequences further showed that 45 of the 48 taxa retained positive barcode gaps, supporting the utility of the library for identifying most taxa while also identifying a small number of lineages that require further study. Equally important, the numt-related cases demonstrate that reference-library quality depends not only on correct specimen identification but also on verification that the sequence itself represents an authentic mitochondrial COI. Stop codon screening alone is insufficient; suspicious records should be evaluated using multiple lines of evidence and clearly flagged until independently confirmed. Thus, the broader value of this dataset lies in the combination of regional taxonomic coverage, voucher-based traceability, public accessibility, and explicit quality control of the data. The continued addition of individuals and geographic populations will improve estimates of intraspecific variation. However, the present library already provides a curated foundation for molecular identification and biodiversity research on Taiwan’s non-volant terrestrial mammals.

## Additional information

### Artificial Intelligence (AI) use

Regarding the use of AI in the preparation of this manuscript, the authors declare the following:

During the preparation of this manuscript, the authors used ChatGPT to obtain suggestions for improving the English grammar and phrasing in the initial draft. PaperPal was used to receive recommendations for manuscript formatting and stylistic refinements. Following the use of these tools, the authors reviewed and edited the content as needed and took full responsibility for the accuracy and integrity of the final published work.

### Author ORCIDs

Y.-J. Chen https://orcid.org/0009-0008-9757-5105

S.-W. Chang https://orcid.org/0000-0001-8431-5162

N.-H. Jang-Liaw https://orcid.org/0000-0002-3481-2045

### Data availability

All of the data of this study are available under the project “Terrestrial Mammals of Taiwan (except Chiroptera); TPZMM” in BOLD. (https://doi.org/10.5883/DS-TPZMM).

## Supplementary materials

Supplementary material 1 Detailed metadata for all specimens and sequences are provided in this study Data type xlsx Explanation note The table includes species names, species numbers, sample information, voucher or tissue-archive records, sampling locality codes, sequence information, BOLD Process IDs, and other traceability metadata. The sampling locality codes correspond to the map shown in Fig. 1. https://binary.pensoft.net/file/1789022This dataset is made available under the Open Database License (http://opendatacommons.org/licenses/odbl/1.0/). The Open Database License (ODbL) is a license agreement intended to allow users to freely share, modify, and use this Dataset while maintaining this same freedom for others, provided that the original source and author(s) are credited. Yen-Jean Chen, Yi-Lun Chiang, Meng-Jou Chi, Shih-Wei Chang, Nian-Hong Jang-Liaw

Supplementary material 2 Public GenBank sequences of Taiwanese non-volant terrestrial mammals were incorporated into the expanded Taiwan COI dataset Data type xlsx Explanation note The table lists the 75 additional public sequences used in this study, including accession numbers, taxonomic names adopted in the present study, original database names when different, locality or source information when available, and source references where applicable. https://binary.pensoft.net/file/1789023This dataset is made available under the Open Database License (http://opendatacommons.org/licenses/odbl/1.0/). The Open Database License (ODbL) is a license agreement intended to allow users to freely share, modify, and use this Dataset while maintaining this same freedom for others, provided that the original source and author(s) are credited. Yen-Jean Chen, Yi-Lun Chiang, Meng-Jou Chi, Shih-Wei Chang, Nian-Hong Jang-Liaw

Supplementary material 3 Species-level K2P distance and barcode-gap comparisons between the original and expanded Taiwan COI datasets Data type xlsx Explanation note The original dataset includes 157 newly generated sequences, whereas the expanded Taiwan dataset includes these 157 sequences plus 75 public GenBank sequences. K2P distances were calculated in MEGA 12.1, and species-level summaries and barcode-gap metrics were calculated from MEGA-generated distance matrices in Microsoft Excel. https://binary.pensoft.net/file/1789024This dataset is made available under the Open Database License (http://opendatacommons.org/licenses/odbl/1.0/). The Open Database License (ODbL) is a license agreement intended to allow users to freely share, modify, and use this Dataset while maintaining this same freedom for others, provided that the original source and author(s) are credited. Yen-Jean Chen, Yi-Lun Chiang, Meng-Jou Chi, Shih-Wei Chang, Nian-Hong Jang-Liaw

Supplementary material 4 Suspected numts, stop-codon-containing sequences, and putative COI-like pseudogene sequences were identified in the COI dataset Data type xlsx Explanation note Sequences were flagged based on one or more lines of evidence, including stop-codon detection in the 527-bp trimmed alignment, anomalous NJ placement, barcode-gap context, and/or full-length sequence inspection. This table should be interpreted as sequence-quality and numt-screening metadata, not as direct taxonomic evidence. https://binary.pensoft.net/file/1789025This dataset is made available under the Open Database License (http://opendatacommons.org/licenses/odbl/1.0/). The Open Database License (ODbL) is a license agreement intended to allow users to freely share, modify, and use this Dataset while maintaining this same freedom for others, provided that the original source and author(s) are credited. Yen-Jean Chen, Yi-Lun Chiang, Meng-Jou Chi, Shih-Wei Chang, Nian-Hong Jang-Liaw

Supplementary material 5 Complete Neighbor-Joining (NJ) tree for the expanded Taiwan COI dataset showing all 232 tips, including 157 newly generated sequences and 75 public GenBank sequences Data type xlsx Explanation note Public sequences are indicated by blue text; red stars denote stop-codon-containing sequences and/or putative numts. Taxa 10, 11, and 45 are highlighted for further inspection. The scale bar represents a 0.02 K2P distance. https://binary.pensoft.net/file/1789026This dataset is made available under the Open Database License (http://opendatacommons.org/licenses/odbl/1.0/). The Open Database License (ODbL) is a license agreement intended to allow users to freely share, modify, and use this Dataset while maintaining this same freedom for others, provided that the original source and author(s) are credited. Yen-Jean Chen, Yi-Lun Chiang, Meng-Jou Chi, Shih-Wei Chang, Nian-Hong Jang-Liaw
